# Supplementary material for: Visualizing PIEZO1 localization and activity in hiPSC-derived single cells and organoids with HaloTag technology
Source: Nat Commun. 2025 Jul 1;16:5556. doi: 10.1038/s41467-025-59150-1 (PMC12217361; doi:10.1038/s41467-025-59150-1)
Supplement: Supplementary file 2 — Reporting Summary [file 41467_2025_59150_MOESM2_ESM.pdf]

nature portfolio

Corresponding author(s): Medha M. Pathak

Last updated by author(s): 03/20/2025

Reporting Summary

Nature Portfolio wishes to improve the reproducibility of the work that we publish. This form provides structure for consistency and transparency in reporting. For further information on Nature Portfolio policies, see our Editorial Policies and the Editorial Policy Checklist.

Statistics

For all statistical analyses, confirm that the following items are present in the figure legend, table legend, main text, or Methods section.

n/a	Confirmed	
☐	☒	The exact sample size (*n*) for each experimental group/condition, given as a discrete number and unit of measurement
☒	☐	A statement on whether measurements were taken from distinct samples or whether the same sample was measured repeatedly
☐	☒	The statistical test(s) used AND whether they are one- or two-sided<br>*Only common tests should be described solely by name; describe more complex techniques in the Methods section.*
☒	☐	A description of all covariates tested
☐	☒	A description of any assumptions or corrections, such as tests of normality and adjustment for multiple comparisons
☐	☒	A full description of the statistical parameters including central tendency (e.g. means) or other basic estimates (e.g. regression coefficient) AND variation (e.g. standard deviation) or associated estimates of uncertainty (e.g. confidence intervals)
☐	☒	For null hypothesis testing, the test statistic (e.g. *F*, *t*, *r*) with confidence intervals, effect sizes, degrees of freedom and *P* value noted<br>*Give P values as exact values whenever suitable.*
☒	☐	For Bayesian analysis, information on the choice of priors and Markov chain Monte Carlo settings
☒	☐	For hierarchical and complex designs, identification of the appropriate level for tests and full reporting of outcomes
☐	☒	Estimates of effect sizes (e.g. Cohen's *d*, Pearson's *r*), indicating how they were calculated

Our web collection on statistics for biologists contains articles on many of the points above.

Software and code

Policy information about availability of computer code

Data collection	No new software was developed for data acquisition.
Data analysis	No novel software tools were developed. Custom analysis code used has been deposited on Github at https://github.com/Pathak-Lab/PIEZO1-LocalizationTools and at https://github.com/abcucberkeley/piezo1_analysis_pipeline.

For manuscripts utilizing custom algorithms or software that are central to the research but not yet described in published literature, software must be made available to editors and reviewers. We strongly encourage code deposition in a community repository (e.g. GitHub). See the Nature Portfolio guidelines for submitting code & software for further information.

Data

Policy information about availability of data

All manuscripts must include a data availability statement. This statement should provide the following information, where applicable:

- Accession codes, unique identifiers, or web links for publicly available datasets
- A description of any restrictions on data availability
- For clinical datasets or third party data, please ensure that the statement adheres to our policy

Methods, representative movie files, and supplementary information are included in the manuscript. Source data for graphs in the main figures are available as a downloadable Supplementary Data file. We have included data repositories for raw data corresponding to Supplementary Movies in Zenodo and Dryad (see Data Availability statement for links). Complete raw datasets, including source images and analyzed trajectories, included in this study can also be requested from the corresponding author.

Human research participants

Policy information about studies involving human research participants and Sex and Gender in Research.

Reporting on sex and gender	N/A
Population characteristics	N/A
Recruitment	N/A
Ethics oversight	All research involving human stem cells was approved by the University of California, Irvine Human Stem Cell Research Oversight Committee; Stem cell lines used had no patient identifiers. Animal studies were approved by the Institutional Animal Care and Use Committee of University of California at Irvine.

Note that full information on the approval of the study protocol must also be provided in the manuscript.

Field-specific reporting

Please select the one below that is the best fit for your research. If you are not sure, read the appropriate sections before making your selection.

☒ Life sciences ☐ Behavioural & social sciences ☐ Ecological, evolutionary & environmental sciences

For a reference copy of the document with all sections, see nature.com/documents/nr-reporting-summary-flat.pdf

Life sciences study design

All studies must disclose on these points even when the disclosure is negative.

Sample size	We collected data from hundreds of individual PIEZO1 puncta recorded from dozens of fields of views from at least 3 independent experiments (N numbers are indicated in figure legends and Methods). This information is included in the figures and figure legends for each figure.
Data exclusions	Not applicable.
Replication	Figure legends and Methods section describe replicate information.
Randomization	Not applicable.
Blinding	To avoid bias, data was analyzed by automated analysis pipelines wherever possible. During automated analyses, parameters were chosen based on objective properties and applied across the board.

Reporting for specific materials, systems and methods

We require information from authors about some types of materials, experimental systems and methods used in many studies. Here, indicate whether each material, system or method listed is relevant to your study. If you are not sure if a list item applies to your research, read the appropriate section before selecting a response.

Materials & experimental systems

n/a	Involved in the study
☐	☒ Antibodies
☐	☒ Eukaryotic cell lines
☒	☐ Palaeontology and archaeology
☐	☒ Animals and other organisms
☒	☐ Clinical data
☒	☐ Dual use research of concern

Methods

n/a	Involved in the study
☒	☐ ChIP-seq
☒	☐ Flow cytometry
☒	☐ MRI-based neuroimaging

Antibodies

Antibodies used

The primary antibodies used to complete the work are as follows: Mouse anti-N-cadherin BD Biosciences Cat# 610920 1:500 (0.5 µg/ml), mouse anti-Nestin R&D Systems Cat# MAB1259, 1:500 (0.5 µg/ml), rabbit anti-Sox2 Millipore Cat# AB5603 1:500 (2 µg/ml), goat anti-Sox2 R&D Systems Cat# AF2018 1:500 (5 µg/ml), anti-VE Cadherin, rabbit, Abcam ab33168 1:500 (2 µg/ml), mouse anti-CD31 Agilent Technologies Cat# M082329-2 1:500 (0.4 ug/ml), mouse anti-PAXILLIN Millipore Cat# 05-41 1:200 (1 µg/ml), rabbit anti-KERATIN14 Biolegend Cat# 05301 1:100 (10 µg/ml).

The secondary antibodies used to complete this work are as follows: Goat anti-Rabbit IgG Invitrogen Cat# A32731 1:500 (4 µg/ml), goat anti-Mouse IgG Invitrogen Cat# A32727, 1:500 (4 µg/ml), donkey anti-Goat IgG Invitrogen Cat# A21432 1:500 (4 µg/ml). See Supplementary Table 1 for more information.

Validation

Antibodies used in these studies are commercially available and have been readily tested. Necessary control experiments are included in the supplemental data.

Eukaryotic cell lines

Policy information about cell lines and Sex and Gender in Research

Cell line source(s)

WTC-11 human induced pluripotent stem cell line (male, 30 years old) was acquired through Corriell.

Authentication

Human induced pluripotent stem cells were karyotyped using KaryoStat (Applied Biosystems) Analysis to ensure genomic stability. Pluripotency was checked by immunocytochemical staining with pluripotency markers NANOG, OCT4, SOX2, SSEA-4, TRA1-60, TRA1-81.

Mycoplasma contamination

Mycoplasma tests were performed and the results were negative.

Commonly misidentified lines
(See ICLAC register)

We did not use any eukaryotic cell lines from the ICLAC table of commonly misidentified lines.

Animals and other research organisms

Policy information about studies involving animals; ARRIVE guidelines recommended for reporting animal research, and Sex and Gender in Research

Laboratory animals

The Piezo1-tdTomato mice (JAX stock 029214) were used in this study. Mice were housed in standard conditions at UCI's university laboratory animal resources facility.

Wild animals

No wild animals were used to complete this study.

Reporting on sex

Sex was not considered for study design .

Field-collected samples

No field collected samples were used to complete this study.

Ethics oversight

All animal experiments for this study were approved by the Institutional Animal Care and Use Committee (IACUC) at University of California, Irvine.

Note that full information on the approval of the study protocol must also be provided in the manuscript.
