# Supplementary material for: Visualizing PIEZO1 localization and activity in hiPSC-derived single cells and organoids with HaloTag technology
Source: Nat Commun. 2025 Jul 1;16:5556. doi: 10.1038/s41467-025-59150-1 (PMC12217361; doi:10.1038/s41467-025-59150-1)
Supplement: Supplementary file 3 — Description of additional supplementary files [file 41467_2025_59150_MOESM3_ESM.pdf]

Description of Additional Supplementary Files

Supplementary Movie 1. PIEZO1 puncta imaged in hiPSC-derived PIEZO1-HaloTag endothelial cells. A representative PIEZO1-HaloTag endothelial cell labeled with the JF646 HTL and imaged using TIRF microscopy for a duration of 2 minutes at 10 fps.

Supplementary Movie 2. PIEZO1 puncta imaged in hiPSC-derived PIEZO1-HaloTag keratinocytes. A representative PIEZO1-HaloTag keratinocyte labeled with the JF646 HTL and imaged using TIRF microscopy for a duration of 2 minutes at 10 fps.

Supplementary Movie 3. PIEZO1 puncta imaged in hiPSC-derived PIEZO1-HaloTag neural stem cells. A representative PIEZO1-HaloTag neural stem cell labeled with the JF646 HTL and imaged using TIRF microscopy for a duration of 2 minutes at 10 fps.

Supplementary Movie 4. Localized enrichment of PIEZO1 at the rear of migrating NSC. A representative migrating PIEZO1-HaloTag neural stem cell labeled with the JF646 HTL and imaged using TIRF microscopy for 2 minutes at 10 fps.

Supplementary Movie 5. Tracking of PIEZO1-HaloTag puncta in endothelial cells. PIEZO1-HaloTag endothelial cell imaged at 10 fps for 10 s. Trajectories were extracted using the protocol detailed in the Methods section, "Analysis of PIEZO1-HaloTag Puncta Diffusion Based on TIRF Imaging."

Supplementary Movie 6. PIEZO1-HaloTag endothelial cells labeled with JF646-BAPTA HaloTag ligand. TIRF images of JF646-BAPTA labeled PIEZO1-HaloTag and PIEZO1-HaloTag Knockout endothelial cells. Rightmost video shows a PIEZO1-HaloTag endothelial cell treated with 2 μM Yoda1. Videos were acquired at a frame rate of 100 fps.

Supplementary Movie 7. Flickers from a trapped DMSO treated PIEZO1-HaloTag JF646-BAPTA puncta. TIRF video of a trapped punctum from a PIEZO1-HaloTag endothelial cell(left) treated with vehicle control DMSO. Green dot indicates centroid of trajectory and green box indicates 3x3 pixel region used to generate the fluorescence intensity trace populating on the right. Data were acquired at a frame rate of 200 fps

Supplementary Movie 8. Flickers from a trapped 2 μM of Yoda1 treated PIEZO1-HaloTag JF646-BAPTA puncta. TIRF video a trapped punctum from a PIEZO1-HaloTag endothelial cell (left) treated with 2 μM of Yoda1. Green dot indicates centroid of trajectory and green box indicates 3x3 pixel region used to generate the fluorescence intensity trace populating on the right. Data were acquired at a frame rate of 200 fps

Supplementary Movie 9. Simultaneous imaging of PIEZO1-HaloTag JF646-BAPTA puncta mobility and activity. TIRF video of PIEZO1-HaloTag endothelial mobile puncta (left). Green dot indicates centroid of trajectory and the green line shows the trajectory of the puncta movement used to generate the fluorescence intensity trace populating on the right. Data were acquired at a frame rate of 200 fps

Supplementary Movie 10. 3D Visualization of actin and PIEZO1-HaloTag in MNR. PIEZO1-HaloTag in MNRs labeled with JF635 HTL imaged using AO-LLSM and visualized using both 2D orthoslices and 3D volumetric views. The video shows actin (white), denoised nuclei (cyan), PIEZO1-HaloTag (green, raw data; magenta, computationally detected PIEZO1 puncta), followed by a 3D local density map of the PIEZO1-HaloTag detections color-coded for the total number of detected PIEZO1-HaloTag puncta within a 3.6 μm search radius or 200 μm3 search volume centered around each detected PIEZO1 puncta.
